# Digital concepts in post-stroke care pathways: adding value and minimizing burden

**DOI:** 10.1186/s42466-021-00150-z

**Published:** 2021-09-09

**Authors:** Christoph Schöbel, Martin Köhrmann

**Affiliations:** 1 Center for Sleep- and Telemedicine, Ruhrlandklinik – West German Lung Center, Universitätsmedizin Essen, Tüschener Weg 40, 45239 Essen, Germany; 2 Department of Neurology and Center for Translational Neuro- and Behavioral Sciences (C-TNBS), University Hospital Essen, Hufelandstrasse 55, 45147 Essen, Germany; 3 Interdisciplinary Working Group on Telemedicine, Universitätsmedizin Essen, Essen, Germany

In their manuscript “The PostStroke Manager—combining mobile, digital and sensor-based technology with personal assistance: protocol of the feasibility study” Michalski et al. describe an innovative and intersectoral care pathway for post-stroke patients integrating telemedical approaches.

Indeed, new technologies can help to improve patient management after acute medical emergencies or in chronic diseases. For example, randomized controlled trials have shown that the telemedical management can lead to significantly reduced mortality and re-hospitalisation rates in patients with chronic heart failure [1, 2].

The pathway for post-stroke management is still a matter of perceived weakness and intense research. Studies so far have focused on structured, repeated in person follow-ups with strict goals for key surrogate parameters of secondary prevention such as LDL-levels, blood pressure control and physical activity [3, 4]. Including digital technology into such a structured post-stroke pathway is promising. However, integrating such digital approaches should not only focus on clinical aspects but need to investigate care-relevant aspects as well. This should include analyzes not only on financial aspects but also consumption of other i.e. human resources of the health care system—both of which are precious and restricted. Consequentially, analyzes on these points are a prerequisite for funding on innovative care by the Federal Joint Committee (Gemeinsamer Bundesausschuss, GBA) in Germany. Primary data collected in the project are often evaluated together with secondary data from statutory health insurances.

To show feasibility of a digital approach in a trial is just a first step. What would be the use of a feasible innovative care concept if the effort required to implement it in clinical daily routine would be disproportionately high leading to a perceived burden rather than a relief? A major concern is that such structured care-pathways, especially those combining conventional in-person follow-up with additional digital methods, may provide overtreatment to patients without the need of further support while still not reaching those with needs. This problem may even be accentuated by the digital, telemedical approach because the usage of such media is often pronounced in more organized, digitally educated, younger and healthier subpopulations. Research should address intersectoral and multiprofessional care components in a needs-oriented manner. Taking this into account digital care has on the other side the potential to provide a stepped care approach with the right level of care for the individual patients instead of a “one size fits all” approach.

A key safety issue is the validity and reliability of data generated by digital devices in studies. It is mandatory that all digital components providing key data are derived from technology certified as medical device in accordance with the regulatory requirements of the European Medical Device Regulation (MDR). The same holds true for data protection of digital solutions. Failure in these aspects may not only lead to direct safety issues for the individual patient but may also jeopardize the acceptance of the digital approach in total.

In the end, besides proven feasibility as well as efficacy, innovative care concepts can only reach clinical reality if all involved stakeholders (patients, physicians, service providers etc.) experience an added value compared to previous approaches. In addition to the classical clinical endpoints, evidence-generating studies should therefore examine patient-related outcome (PROMs) or experience measurements (PREMs). Even if these endpoints are often seen as “soft factors” within the medical community, they are decisive factors in whether innovative care concepts will proof practicability in clinical daily routine. Only sufficient evidence including the above-mentioned aspects can be the basis for implementing innovative concepts into standard care with reimbursement by health insurances.
